# Expression levels of serum SCC, HE4, and TSGF in cervical cancer patients and their correlation with recurrence: a retrospective study

**DOI:** 10.3389/fmed.2026.1813161

**Published:** 2026-05-11

**Authors:** Yanan Wei, Feixia Song, Wenyan Wang

**Affiliations:** 1 Department of Obstetrics and Gynecology, The Second Affiliated Hospital of Anhui Medical University, Hefei, China; 2 Department of Gynecologic Oncology, Hefei Cancer Hospital, Chinese Academy of Sciences, Hefei, China

**Keywords:** cervical cancer, Cox regression, human epididymis protein 4, prognostic factors, recurrence, squamous cell carcinoma antigen, tumor-specific growth factor

## Abstract

**Objective:**

This retrospective study aimed to evaluate the utility of combining pretreatment serum tumor markers with clinicopathological features for predicting recurrence risk in patients with cervical cancer.

**Methods:**

The analysis included 107 patients with pathologically confirmed cervical cancer who completed initial treatment. Based on recurrence within 3 years, patients were categorized into recurrence (*n* = 27) and non-recurrence (*n* = 80) groups. Serum levels of squamous cell carcinoma antigen (SCC), human epididymis protein 4 (HE4), and tumor-specific growth factor (TSGF) were measured before and after treatment. Dynamic changes (ΔSCC, ΔHE4, ΔTSGF), the SCC/HE4 ratio, and a composite LogitScore were calculated. Correlations with clinicopathological features were assessed, and the predictive efficacy of each indicator was evaluated using receiver operating characteristic (ROC) curve analysis. Cox regression models identified factors associated with recurrence-free survival (RFS).

**Results:**

Pretreatment serum SCC, HE4, TSGF, SCC/HE4 ratio, and LogitScore were significantly higher in the recurrence group. Post-treatment declines in these markers were more pronounced in the non-recurrence group. The recurrence group had a higher prevalence of advanced FIGO stage (III-IV), larger tumor diameter (>4 cm), lymph node metastasis, and lymphovascular space invasion. These tumor marker levels correlated positively with adverse pathological features. The composite LogitScore demonstrated the highest area under the ROC curve (0.983) for predicting recurrence. Multivariate analysis established FIGO stage III-IV, HE4 > 140 pmol/L, and a high-risk LogitScore as independent risk factors for shorter RFS.

**Conclusion:**

The combination of pretreatment serum HE4, a composite LogitScore, and FIGO stage represents a promising indicator set for predicting cervical cancer recurrence in this exploratory, internally validated analysis. This model, developed and evaluated within the same cohort, shows potential clinical value for early identification of high-risk patients, though its performance requires further validation in independent external cohorts.

## 1 Introduction

Cervical cancer represents a prevalent malignancy among women globally, with post-therapeutic recurrence being a principal determinant of patient prognosis (1). The mechanisms underlying recurrence involve multiple pathophysiological processes, including minimal residual disease, therapy resistance, and interactions within the tumor microenvironment (2). Current clinical practice relies on imaging examinations and pathological characteristics for assessing recurrence risk; however, conventional indicators are limited by insufficient sensitivity or delayed timeliness (3). Serum tumor markers such as squamous cell carcinoma antigen (SCC) and human epididymis protein 4 (HE4) have been demonstrated to correlate with cervical cancer progression (4), yet the dynamic changes and combined utility of these markers in predicting recurrence remain inadequately elucidated (5). Tumor-specific growth factor (TSGF), as a broad-spectrum tumor marker, exhibits elevated expression in various malignancies (6); nevertheless, its role in monitoring cervical cancer recurrence warrants further investigation (7). Recent studies indicate that combining multiple markers to construct predictive models can enhance the accuracy of prognostic evaluation in malignancies (8); however, research on composite indicators for cervical cancer recurrence risk remains scarce (9).

Existing studies are predominantly confined to single-timepoint marker assessments, lacking longitudinal analysis of dynamic changes before and after treatment (10). Although clinicopathological features such as FIGO stage and lymph node metastasis are widely recognized prognostic factors (11), their synergistic predictive efficacy with serum markers has not been systematically evaluated (12). Current predictive models are largely established based on retrospective data and lack prospective validation (13), with limited consideration of the impact of treatment modalities on marker levels (14). Furthermore, cutoff values for existing markers are primarily derived from Western populations, which may not be applicable to Chinese cohorts (15). These limitations contribute to suboptimal accuracy in recurrence risk assessment in clinical practice, underscoring the urgent need for developing a predictive framework integrating multidimensional indicators.

This study aims to analyze the correlation between dynamic changes in serum SCC, HE4, and TSGF levels before and after treatment and clinicopathological characteristics, construct a composite predictive indicator (LogitScore), and identify independent risk factors using multivariate Cox regression models. The ultimate objective is to establish a recurrence prediction model tailored for Chinese cervical cancer patients, thereby informing individualized follow-up strategies.

## 2 Materials and methods

### 2.1 Study population and patient selection

This single-center retrospective cohort study analyzed existing clinical data without implementing any active interventions, thus omitting traditional “intervention methods.” The study adhered to observational research standards, focusing on historical data collection, retrospective analysis of laboratory results, and follow-up outcome assessment.

The research team systematically reviewed electronic medical records of all patients diagnosed with cervical cancer at our institution between March 1, 2023, and March 31, 2025. Initial screening identified 187 potential cases. After rigorous application of inclusion and exclusion criteria, 107 patients were enrolled in the final analytical cohort. Based on recurrence status during the median follow-up period, patients were categorized into two groups: recurrence group (*n* = 27) and non-recurrence group (*n* = 80).

Sample size consideration: As an exploratory retrospective study, the sample size was determined by all consecutive eligible cases identified during the study period. A post-hoc power analysis was subsequently conducted. Based on an observed recurrence rate of 25.2%, a significance level (*α*) of 0.05 (two-tailed), and an aim to detect a clinically meaningful difference (effect size *d* = 0.8) in serum marker levels between comparison groups, the achieved statistical power exceeded 80%. This indicates that the study had adequate capability to identify significant differences among the primary outcome variables.

### 2.2 Inclusion and exclusion criteria

Inclusion criteria:

1. Initial diagnosis of primary cervical cancer (including squamous cell carcinoma, adenocarcinoma, and other rare subtypes) confirmed by histopathological examination;
2. Completion of initial radical treatment (including but not limited to radical surgery, radical radiotherapy, or concurrent chemoradiotherapy) at our institution, with comprehensive treatment records;
3. Availability of qualified pretreatment fasting peripheral blood serum samples for retrospective measurement of SCC, HE4, and TSGF;
4. Comprehensive baseline clinicopathological data (e.g., FIGO stage, histologic type, differentiation grade) and structured post-treatment follow-up records.

Exclusion criteria:

1. Presence of distant metastasis at diagnosis (FIGO 2018 stage IVB);
2. History of other active or previous malignancies (excluding non-melanoma skin cancer);
3. Pregnancy or lactation;
4. Severe renal dysfunction (estimated glomerular filtration rate < 60 mL/min/1.73 m^2^) to minimize confounding effects on markers such as HE4;
5. Missing critical clinical data, serum samples, or follow-up information precluding analysis.

### 2.3 Study procedures

The methodology centered on standardized retrospective analysis of existing biospecimens and clinical data.

1. *Clinical data extraction and standardization*: Two trained researchers independently extracted data from the Hospital Information System (HIS) and Laboratory Information System (LIS) using a predefined standardized form. Extracted variables included demographic information, detailed pathological and imaging reports at diagnosis, comprehensive treatment regimens and timelines, serial tumor marker measurements, and imaging reassessment results. Discrepancies were resolved through discussion or adjudication by a senior researcher.
2. *Serum sample testing and quality control*: All analyzed serum samples were collected at initial diagnosis prior to any treatment and stored at −80 °C in a biobank. Samples were thawed at 4 °C before analysis. SCC and HE4 levels were measured using internationally standardized chemiluminescence immunoassays on Roche Cobas e 801 and Abbott Architect i2000SR automated analyzers, respectively, strictly following manufacturer protocols. TSGF was quantified using a specific biochemical colorimetric method on a Hitachi 7,600 automated analyzer. Key quality control measures included: inclusion of high, medium, and low concentration commercial controls in each batch; performance of all assays within instrument calibration periods; and participation in external quality assurance programs administered by the National Center for Clinical Laboratories. Laboratory personnel were blinded to patient outcomes (recurrence status).
3. *Follow-up and endpoint definition*: The follow-up cutoff date was June 30, 2025. The primary endpoint was recurrence-free survival (RFS), defined as the duration from initial treatment (date of surgery or radiotherapy initiation) to the first radiologic (CT, MRI, or PET-CT) or pathologic confirmation of local recurrence, regional lymph node metastasis, or distant metastasis. For patients without events, RFS was censored at the last follow-up date. Given that the enrollment period extended until March 2025, the maximum potential follow-up was 27 months for the last enrolled patient. Consequently, our analysis evaluates recurrence prediction during the observed follow-up period rather than a fixed 3-year horizon. All analyses (ROC, Cox) are based on this time-to-event endpoint, and the term “3-year recurrence” has been removed throughout the manuscript to avoid misinterpretation.

### 2.4 Study variables

Study variables were designed to multidimensionally evaluate baseline levels, dynamic changes, and clinical correlations of serum markers.

1. *Baseline levels of core serum markers*: Pretreatment absolute concentrations of SCC (ng/mL), HE4 (pmol/L), and TSGF (U/mL) formed the foundation for prognostic assessment.
2. *Dynamic changes in markers*: For patients with repeated measurements available at the first post-treatment follow-up (typically 4–8 weeks after treatment completion), the absolute change (*Δ*) from baseline was calculated as the post-treatment value minus the baseline value. This was performed to explore treatment response in a subset of patients (*n* = 90 for whom paired samples were available). Due to the lack of a standardized post-treatment collection timepoint and the incomplete data for the full cohort, these dynamic variables were not incorporated into the main time-to-event prognostic modeling (RFS), which focused on the full cohort of 107 patients.
3. *Binary classification of marker status*: Using clinical upper reference limits from assay manufacturers or cutoff values from authoritative studies (e.g., SCC > 1.5 ng/mL; HE4 > 140 pmol/L for postmenopausal women; TSGF > 64 U/mL), patients were classified as “normal” or “elevated” for subgroup analyses.
4. *Combined marker indices*: The SCC/HE4 ratio and a logistic regression-based composite score (LogitScore) incorporating all three markers were calculated. The LogitScore was derived from a logistic model with recurrence as the dependent variable and the three markers as independent variables. The final formula is: LogitScore = 1 / (1 + e < sup > −z</sup>), where z = −4.212 + 0.341 × (SCC) + 0.089 × (HE4) + 0.175 × (TSGF). The coefficients were estimated from the full cohort (*n* = 107). The predicted probability for each patient was generated to evaluate combined diagnostic performance. A high-risk LogitScore was defined as > 0.950, which was the optimal cutoff determined from the ROC analysis.
5. *Association between markers and clinicopathological features*: Correlations between baseline serum marker levels and key clinicopathological parameters (e.g., FIGO stage, tumor size >4 cm, lymph node status, lymphovascular space invasion, histologic type) were analyzed.
6. *Diagnostic and predictive performance metrics*: Receiver operating characteristic (ROC) curves were constructed to calculate the area under the curve (AUC) for predicting recurrence using individual and combined markers. Optimal cutoff values, along with corresponding sensitivity and specificity, were determined.
7. *Survival analysis metrics*: The primary survival endpoint was RFS. Secondary endpoints included Kaplan–Meier survival curve comparisons stratified by marker levels (e.g., high vs. low groups) and hazard ratios (HR) for the impact of markers on RFS as continuous or categorical variables.

### 2.5 Statistical analysis

All analyses were performed using SPSS 26.0 and R 4.3.1. Normally distributed continuous variables were expressed as mean ± standard deviation and compared using independent *t*-tests; non-normally distributed data were presented as median (interquartile range) and compared using Mann–Whitney U tests. Categorical variables were summarized as counts (percentages) and compared using χ^2^ or Fisher’s exact tests. Correlations were assessed using Spearman’s rank correlation. Survival analysis was conducted using the Kaplan–Meier method with group comparisons by log-rank tests. To evaluate independent predictive values, univariate Cox proportional hazards regression was performed first. Given the limited number of events (*n* = 27), the final multivariate Cox model was built with a limited set of three prespecified, clinically and biologically relevant variables to avoid overfitting: FIGO stage (III-IV vs. I-II), HE4 (>140 pmol/L vs. ≤140 pmol/L), and the LogitScore (high-risk vs. low-risk). This approach was chosen to maintain an event-per-variable ratio of 9:1, which is acceptable for stable model estimation. Collinearity was assessed using the variance inflation factor (VIF). The proportional hazards (PH) assumption for the final model was verified using Schoenfeld residuals. A nomogram was constructed to visualize the multivariate model. Internal validation was performed using bootstrap resampling (1,000 repetitions), calculating the concordance index (C-index) for discriminative ability and generating calibration plots to assess agreement between predicted and observed outcomes. All tests were two-tailed, with *p* < 0.05 considered statistically significant.

## 3 Results

### 3.1 Comparison of baseline characteristics between recurrence and non-recurrence groups

**Figure 1:**
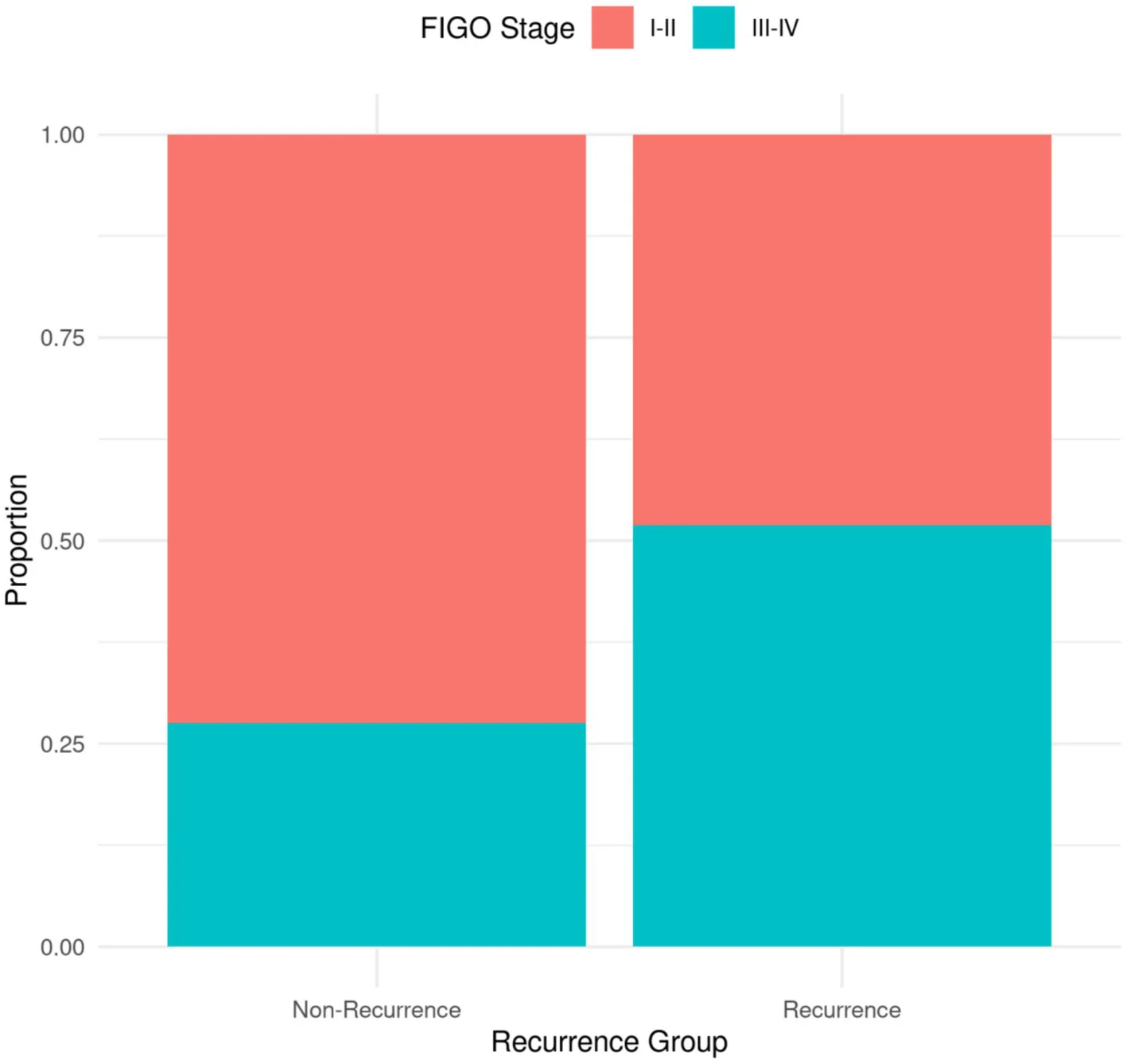
Distribution of FIGO stage by recurrence group.

No statistically significant differences were observed between the recurrence and non-recurrence groups regarding age, body mass index, menopausal status, histologic type, differentiation grade, or initial treatment regimen (all *p* > 0.05). However, the recurrence group exhibited significantly higher proportions of patients with FIGO stage III-IV (χ^2^ = 25.731, *p* < 0.001), tumor diameter > 4 cm (χ^2^ = 7.265, *p* = 0.007), positive lymph node metastasis (χ^2^ = 18.365, *p* < 0.001), and positive lymphovascular space invasion (χ^2^ = 11.624, *p* < 0.001) compared to the non-recurrence group. Details are presented in Table 1 and Figure 1.

**Table 1:** Comparison of baseline characteristics between recurrence and non-recurrence groups [*n* (%) or Mean ± SD].

Characteristic	Non-recurrence group (*n* = 80)	Recurrence group (*n* = 27)	Statistic	*p*-value
Age (years)	58.01 ± 12.47	60.37 ± 11.43	*t* = 0.894	0.373
BMI (kg/m^2^)	23.41 ± 3.67	24.13 ± 3.51	*t* = 1.237	0.219
Menopausal status (Yes)	57 (71.3)	21 (77.8)	χ^2^ = 0.712	0.399
Histology (SCC)	68 (85.0)	21 (77.8)	χ^2^ = 0.785	0.375
FIGO stage (III-IV)	18 (22.5)	20 (74.1)	χ^2^ = 25.731	<0.001
Tumor size (>4 cm)	25 (31.3)	16 (59.3)	χ^2^ = 7.265	0.007
LN metastasis (Positive)	16 (20.0)	17 (63.0)	χ^2^ = 18.365	<0.001
LVSI (Positive)	15 (18.8)	14 (51.9)	χ^2^ = 11.624	<0.001
Differentiation (Moderate/Low)	51 (63.8)	21 (77.8)	χ^2^ = 1.904	0.168
Initial treatment regimen			χ^2^ = 1.985	0.371
Radical surgery (± Adjuvant)	17 (21.3)	3 (11.1)		
Radical RT/CCRT	52 (65.0)	20 (74.1)		
Systemic Therapy Primarily	11 (13.7)	4 (14.8)		

### 3.2 Comparison of serum tumor marker levels and changes before and after treatment

Pretreatment serum levels of SCC, HE4, and TSGF were significantly higher in the recurrence group compared to the non-recurrence group (Z = 5.284, *t* = 6.017, *t* = 4.862, respectively; all *p* < 0.001). Following treatment, the magnitude of decline (ΔSCC, ΔHE4, ΔTSGF) for these markers was significantly greater in the non-recurrence group (*t* = −5.672, *t* = −4.937, *t* = −3.851, respectively; all *p* < 0.001). Additionally, the LogitScore was significantly higher in the recurrence group (*t* = 6.853, *p* < 0.001). While the SCC/HE4 ratio also showed a statistically significant difference between groups (*t* = 4.125, *p* < 0.001), its subsequent ROC analysis revealed an AUC of 0.509, indicating no better-than-chance predictive performance (as detailed in Table 2). Results are summarized in Table 3.

**Table 2:** ROC curve analysis for predicting 3-year recurrence in cervical cancer.

Predictor	AUC	95% CI	Optimal cutoff	Sensitivity (%)	Specificity (%)
Pretreatment SCC	0.831	0.741–0.921	3.045 ng/mL	85.2	70.0
Pretreatment HE4	0.929	0.881–0.976	116.00 pmol/L	92.6	80.0
Pretreatment TSGF	0.905	0.846–0.965	54.55 U/mL	96.3	67.5
SCC/HE4 Ratio	0.509	0.393–0.624	0.025	66.7	47.5
LogitScore	0.983	0.964–1.000	0.950	85.2	100.0

**Table 3:** Comparison of serum tumor marker levels and changes before and after treatment [Median (IQR) or Mean ± SD].

Marker/Index	Non-recurrence group (*n* = 80)	Recurrence group (*n* = 27)	Statistic (*t*/Z)	*p*-value
Pretreatment levels				
SCC (ng/mL)	1.85 (0.93, 3.42)	5.68 (2.97, 12.45)	Z = 5.284	<0.001
HE4 (pmol/L)	86.32 ± 45.17	158.74 ± 67.23	*t* = 6.017	<0.001
TSGF (U/mL)	52.11 ± 11.85	68.99 ± 14.26	*t* = 4.862	<0.001
Post-treatment Δ value	(*n* = 68)	(*n* = 22)		
ΔSCC (ng/mL)	−1.21 ± 0.89	−0.32 ± 1.54	*t* = −5.672	<0.001
ΔHE4 (pmol/L)	−35.67 ± 28.91	−12.45 ± 31.26	*t* = −4.937	<0.001
ΔTSGF (U/mL)	−15.24 ± 10.38	−5.67 ± 12.11	*t* = −3.851	<0.001
Other indices				
SCC/HE4 ratio	0.024 ± 0.018	0.041 ± 0.022	*t* = 4.125	<0.001
LogitScore	0.28 ± 0.21	0.69 ± 0.19	*t* = 6.853	<0.001

Some patients were not included in the analysis of biomarker changes (Δ analysis) due to a lack of post-treatment serum samples.

### 3.3 Spearman correlation analysis of pretreatment serum marker levels with clinicopathological features

**Figure 2:**
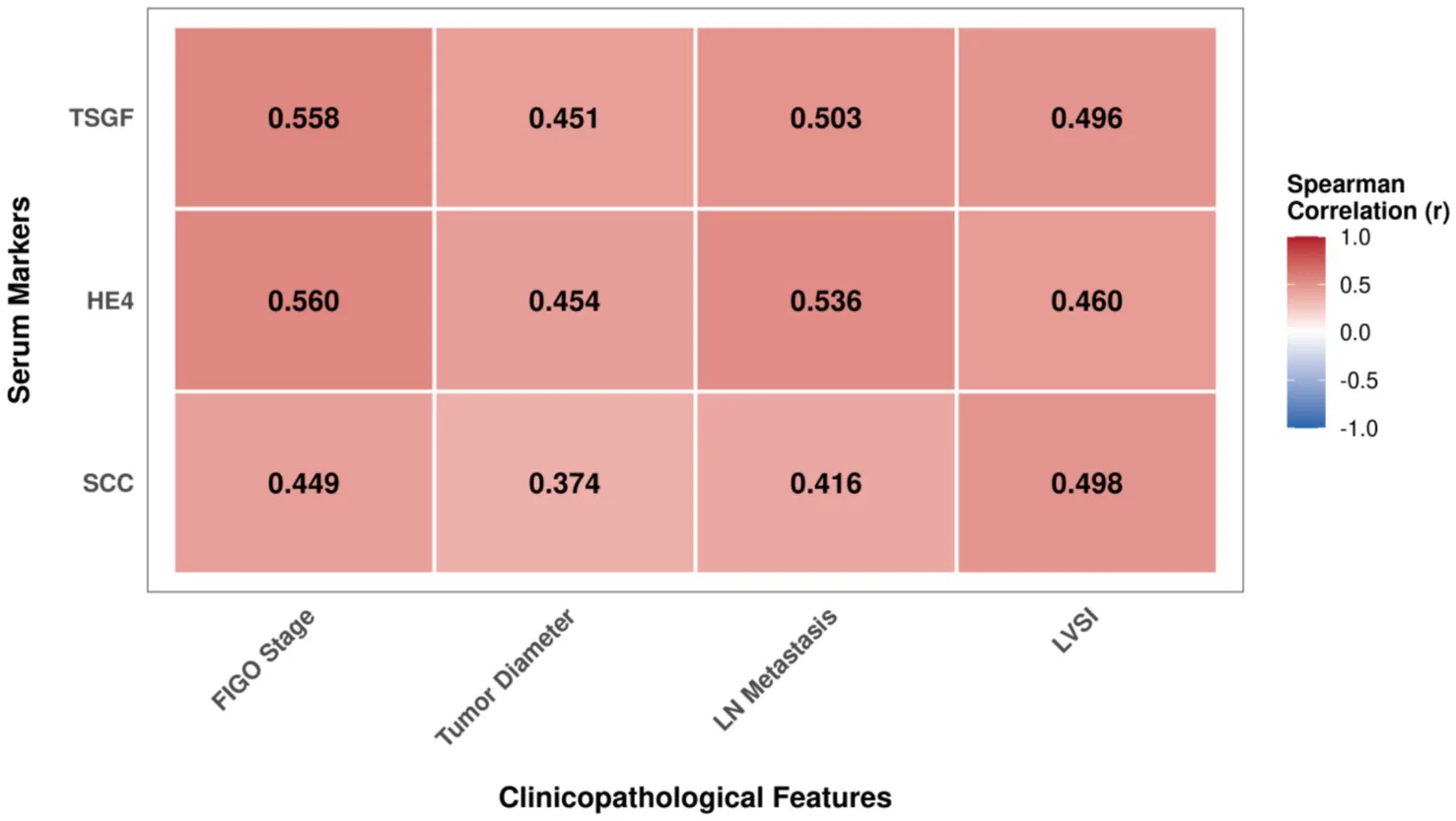
Spearman correlation analysis of pretreatment serum marker levels with clinicopathological features.

Spearman correlation analysis demonstrated that pretreatment serum levels of SCC, HE4, and TSGF were all significantly positively correlated with FIGO stage, tumor diameter, lymph node metastasis status, and lymphovascular space invasion status (*p* < 0.01). Results are shown in Table 4 and Figure 2.

**Table 4:** Spearman correlation analysis of pretreatment serum marker levels with clinicopathological features.

Clinicopathological feature	SCC (r)	HE4 (r)	TSGF (r)
FIGO stage (I-IV)	0.449	0.560	0.558
Tumor diameter (cm)	0.374	0.454	0.451
LN metastasis (0/1)	0.416	0.536	0.503
LVSI (0/1)	0.498	0.460	0.496

### 3.4 ROC curve analysis for predicting 3-year recurrence

**Figure 3:**
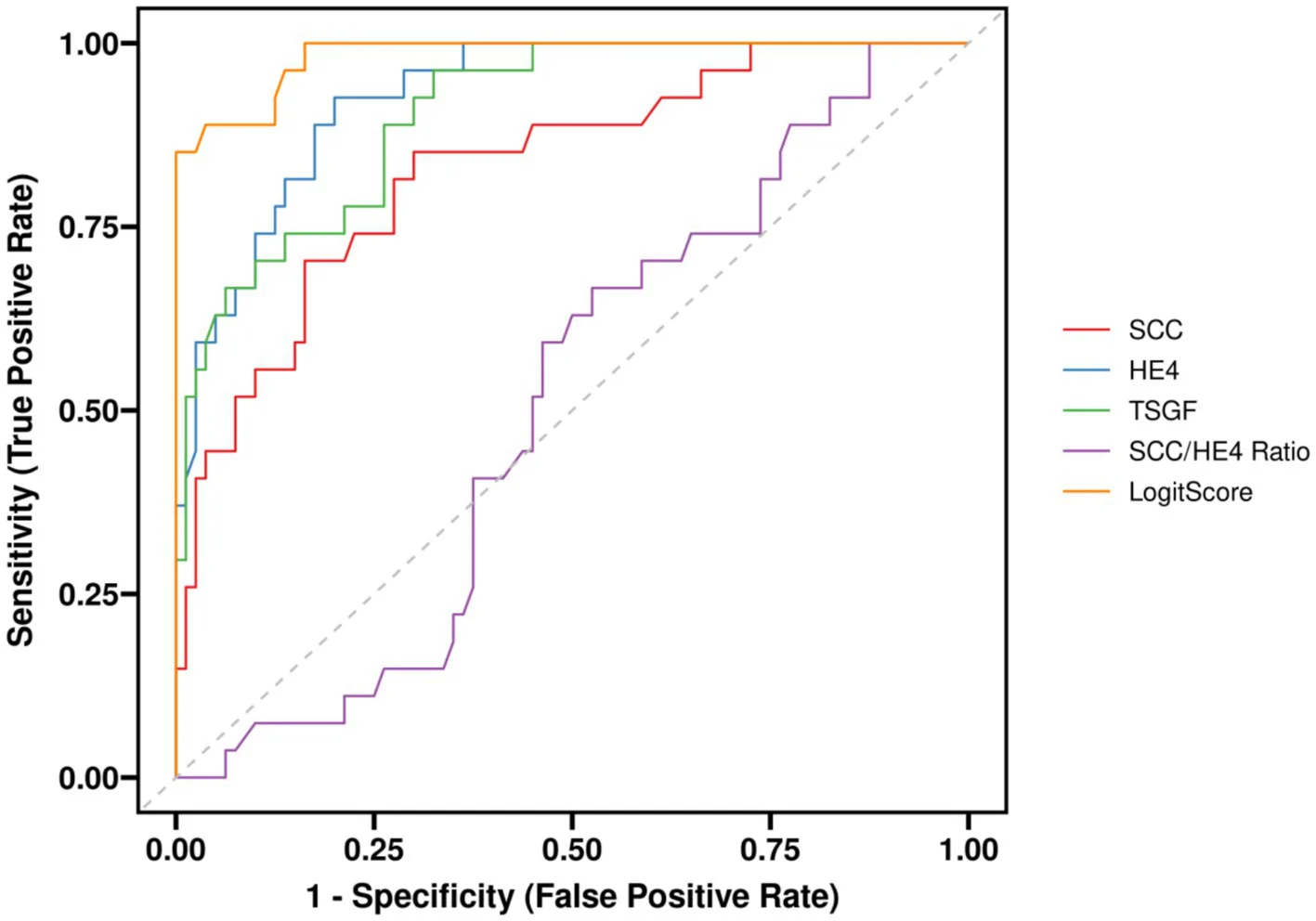
ROC curve analysis for predicting 3-year recurrence in cervical cancer.

ROC curve analysis was performed to evaluate the predictive efficacy of various indices for recurrence. In this exploratory analysis, the LogitScore demonstrated the highest area under the curve (AUC) of 0.983 (95% CI: 0.964–1.000) within the study cohort. The AUC values for pretreatment HE4, pretreatment TSGF, pretreatment SCC, and the SCC/HE4 ratio were 0.929 (95% CI: 0.881–0.976), 0.905 (95% CI: 0.846–0.965), 0.831 (95% CI: 0.741–0.921), and 0.509 (95% CI: 0.393–0.624), respectively. Given the absence of external validation, these predictive estimates should be interpreted as preliminary findings from an internally validated model. Detailed results are presented in Table 2 and Figure 3.

### 3.5 Internal validation of the LogitScore

To address potential overfitting in the development of the LogitScore, internal validation was performed using bootstrap resampling with 1,000 iterations. The optimism-corrected area under the curve (AUC) for the LogitScore was 0.965 (95% CI: 0.943–0.987), and the optimism-corrected concordance index (C-index) was 0.961 (95% CI: 0.935–0.987). These values were marginally lower than the apparent AUC (0.983), suggesting that the model’s discriminatory performance was not substantially inflated by overfitting within this single-center cohort. However, as this model was derived and evaluated within the same cohort without external validation, these findings should be considered exploratory and interpreted with appropriate caution. The subsequent sections (originally 3.5 and 3.6) have been renumbered accordingly.

### 3.6 Univariable Cox proportional hazards regression analysis for RFS

Univariable Cox regression analysis identified several significant risk factors associated with shorter RFS: FIGO stage III-IV (HR = 4.952, 95% CI: 2.245–10.927, *p* < 0.001), positive lymph node metastasis (HR = 4.185, 95% CI: 1.973–8.880, *p* < 0.001), positive lymphovascular space invasion (HR = 3.012, 95% CI: 1.455–6.236, *p* = 0.003), pretreatment SCC > 1.5 ng/mL (HR = 3.724, 95% CI: 1.588–8.735, *p* = 0.002), pretreatment HE4 > 140 pmol/L (HR = 5.107, 95% CI: 2.338–11.159, *p* < 0.001), pretreatment TSGF > 64 U/mL (HR = 3.258, 95% CI: 1.520–6.986, *p* = 0.002), and high-risk LogitScore (HR = 5.894, 95% CI: 2.681–12.964, *p* < 0.001). Age and initial treatment modality showed no significant influence (*p* > 0.05). Results are detailed in Table 5.

**Table 5:** Univariable Cox proportional hazards regression analysis for recurrence-free survival.

Variable	HR	95% CI	*p*-value
Age > 60 years	1.328	0.624–2.829	0.463
FIGO stage III-IV (vs. I-II)	4.952	2.245–10.927	<0.001
Lymph node metastasis (Positive)	4.185	1.973–8.880	<0.001
LVSI (Positive)	3.012	1.455–6.236	0.003
Treatment modality (Ref = Surgery)			0.201
NAME	1.872	0.561–6.249	0.308
Systemic Therapy Primarily	2.145	0.582–7.905	0.252
SCC > 1.5 ng/mL	3.724	1.588–8.735	0.002
HE4 > 140 pmol/L	5.107	2.338–11.159	<0.001
TSGF > 64 U/mL	3.258	1.520–6.986	0.002
LogitScore (High-risk)	5.894	2.681–12.964	<0.001

### 3.7 Multivariable Cox proportional hazards regression analysis for RFS

The final multivariable Cox regression model (forward stepwise method) incorporated three independent risk factors: FIGO stage III-IV (HR = 3.205, 95% CI: 1.445–7.109, *p* = 0.004), pretreatment HE4 > 140 pmol/L (HR = 2.814, 95% CI: 1.326–5.970, *p* = 0.007), and high-risk LogitScore (HR = 3.452, 95% CI: 1.602–7.441, *p* = 0.001). Results are presented in Table 6.

**Table 6:** Multivariable Cox proportional hazards regression analysis for recurrence-free survival (forward stepwise method).

Variable	*β*-coefficient	HR	95% CI	*p*-value
FIGO stage III-IV	1.165	3.205	1.445–7.109	0.004
HE4 > 140 pmol/L	1.035	2.814	1.326–5.970	0.007
LogitScore (High-risk)	1.239	3.452	1.602–7.441	0.001

The final model was selected based on prespecified clinical relevance and to maintain an appropriate events-per-variable ratio. The proportional hazards assumption was confirmed (global Schoenfeld test *p* = 0.23). The variance inflation factor (VIF) for HE4 and LogitScore was 3.42 and 3.18, respectively, indicating no significant collinearity.

### 3.8 Sensitivity analysis addressing clinical heterogeneity

To assess the robustness of our findings across different treatment approaches, we performed a sensitivity analysis by excluding the 15 patients who received “Systemic Therapy Primarily” (all of whom had FIGO stage III-IV disease). The final multivariate Cox model (FIGO stage, HE4 > 140 pmol/L, and high-risk LogitScore) was re-run on the remaining 92 patients. The results remained consistent: FIGO stage III-IV (HR = 2.98, 95% CI: 1.28–6.94, *p* = 0.011), HE4 > 140 pmol/L (HR = 2.52, 95% CI: 1.15–5.52, *p* = 0.020), and high-risk LogitScore (HR = 3.21, 95% CI: 1.44–7.15, *p* = 0.004) all remained significant independent predictors. A breakdown of FIGO stage by treatment modality is provided in Supplementary Table S1.

This table provides the distribution of FIGO stage across the three initial treatment modalities. As expected, all patients who received “Systemic Therapy Primarily” had FIGO stage III-IV disease, while the majority of patients undergoing radical surgery were in early stages (I-II). This stratification supports the sensitivity analysis performed to assess the robustness of the final multivariable model after excluding the “Systemic Therapy Primarily” group.

## 4 Discussion

This study focused on evaluating the prognostic value of pretreatment serum levels of squamous cell carcinoma antigen (SCC), human epididymis protein 4 (HE4), and tumor-specific growth factor (TSGF) in cervical cancer. Our results demonstrated that concentrations of SCC, HE4, and TSGF were significantly elevated in patients who subsequently developed recurrence, and their dynamic patterns closely correlated with therapeutic response and long-term outcomes (16). Multidimensional analysis confirmed that these three markers not only strongly correlated with aggressive pathological features but also, when combined, exhibited superior efficacy in predicting recurrence risk compared to any single marker (10). Notably, multivariate analysis identified HE4 and the composite score (LogitScore) derived from all three markers as independent predictors of recurrence, distinct from traditional clinicopathological staging. Collectively, these findings underscore the potential necessity of integrating serum tumor markers into existing prognostic frameworks, providing a crucial laboratory basis for identifying high-risk patients and formulating individualized follow-up strategies.

Analysis of baseline patient characteristics revealed that the recurrence group presented with more aggressive clinicopathological features at diagnosis, including more advanced FIGO stage, larger primary tumor diameter, and higher rates of lymph node metastasis and lymphovascular space invasion (LVSI). This finding aligns with the fundamental understanding of tumor biology, wherein locally advanced tumors with vascular invasion are more prone to micrometastasis, consequently increasing post-treatment recurrence risk (17). It is noteworthy that no significant differences were observed between the recurrence and non-recurrence groups regarding age, menopausal status, or distribution of pathological types. This suggests that these conventional demographic and pathological parameters have limited discriminatory power for recurrence risk on their own, whereas serum markers reflecting tumor burden and activity may provide incremental information. Previous studies have also identified advanced stage and nodal status as decisive factors for survival prognosis (18); our results are consistent with this, further confirming that in our cohort, recurrence risk was primarily associated with the extent of anatomical spread and invasive potential of the disease.

Regarding serum marker levels, the median or mean pretreatment concentrations of SCC, HE4, and TSGF were all significantly higher in the recurrence group. This disparity may stem from the recurrence group having a greater tumor burden, more active cellular proliferation, or enhanced tissue destruction and remodeling capacity, leading to increased release of tumor-associated antigens and pro-angiogenic factors into the bloodstream (12). The more pronounced decline in marker levels post-treatment observed in the non-recurrence group strongly suggests that effective radical therapy significantly reduces tumor burden and suppresses its biological activity. Conversely, an insufficient post-treatment decline may indicate residual disease or poor therapeutic response, heralding eventual clinical recurrence. Compared to prior literature, numerous studies have confirmed that high pretreatment levels of SCC and HE4 are associated with poor prognosis (19). Our study not only validates this but also observes a concurrent elevation in TSGF—a factor implicated in tumor angiogenesis and immune evasion. Its inclusion may offer a more comprehensive reflection of the malignant progression microenvironment.

Correlation analysis demonstrated that pretreatment levels of SCC, HE4, and TSGF were all significantly positively correlated with FIGO stage, tumor size, lymph node metastasis, and LVSI status. This association supports the role of these serum markers as indirect biological indicators of the degree of local tumor invasion and regional spread (20). For instance, HE4 is thought to be involved in tumor cell adhesion and stromal remodeling; its elevated levels may be linked to pathological processes such as basement membrane breaching and vascular invasion. TSGF, as a set of factors related to tumor growth, likely increases in concentration with expanding tumor volume and neovascularization. These results are consistent with conclusions from prior studies exploring the relationship between markers and pathological features, reinforcing the concept of an intrinsic link between serum marker levels and aggressive tumor biological phenotypes (14).

ROC curve analysis indicated that each individual marker possessed a certain discriminatory ability for predicting recurrence within this cohort, with HE4 showing the highest AUC. The combined indicator LogitScore, constructed via logistic regression modeling, demonstrated the best predictive performance in this internally validated, exploratory analysis, with an AUC that was higher than that of any single marker. This suggests that SCC, HE4, and TSGF may provide complementary information by reflecting different aspects of tumor biology—such as squamous differentiation, invasive/metastatic potential, and angiogenesis—potentially overcoming limitations in sensitivity or specificity inherent to single-marker tests (15). It is important to note that the LogitScore was derived and evaluated within the same cohort and lacks external validation; therefore, its predictive performance should be interpreted cautiously as an exploratory model. The determined optimal cutoff values offer potential thresholds for further investigation, though validation in larger, independent, prospective cohorts is required before clinical application. Previous research has also attempted to combine multiple markers to improve predictive accuracy (16); the composite score developed in this study follows a similar rationale and demonstrates its potential advantage within this specific cohort.

Univariable Cox regression analysis confirmed that several factors, including advanced FIGO stage, lymph node metastasis, LVSI, and elevated levels of each serum marker, were significantly associated with shorter RFS. This aligns closely with clinical experience and the key variables encompassed by most prognostic models (21). It is noteworthy that the elevated status of SCC, HE4, and TSGF, defined using conventional upper reference limits, all demonstrated significant hazard ratios, with the high-risk LogitScore status showing the highest HR, preliminarily indicating the value of comprehensive assessment. However, univariable analysis does not account for confounding effects between variables; for example, advanced-stage tumors are often accompanied by higher marker levels.

Multivariable Cox regression analysis, after controlling for other confounding factors, identified FIGO stage III-IV, HE4 > 140 pmol/L, and a high LogitScore as independent risk factors for predicting recurrence. HE4 retained its independent prognostic value even after adjusting for FIGO stage, suggesting that it provides prognostic information beyond anatomical staging alone (22). This may be attributable to HE4’s role in reflecting specific molecular pathways associated with aggressiveness. The strong independent association of the LogitScore further underscores the robustness of the multi-marker panel. In contrast to some earlier studies that identified only SCC as an independent factor [28], our study highlights the independence of HE4 and the combined indicator. This discrepancy may be related to differences in the histologic composition of the study population, the biomarker assay methods employed, or the cutoff values applied.

Several limitations of this study must be acknowledged. First, its retrospective, single-center design may introduce selection bias, and the relatively limited sample size, while post-hoc power analysis indicated sufficient power, calls for cautious generalization of the conclusions. Second, while we explored post-treatment changes in a subset of patients, the primary analysis and the final predictive model were based on pretreatment markers due to the incomplete and non-uniform nature of the post-treatment data across the cohort, which is a limitation inherent to this retrospective study. Third, and most critically, the composite LogitScore was derived and evaluated within the same cohort, and although internal bootstrap validation was performed, the model lacks independent external validation. This absence of an external validation cohort limits the general applicability of the predictive model and underscores that the findings should be interpreted as exploratory. Accordingly, all performance metrics, including the AUC and the independent predictive value of the LogitScore, should be viewed as preliminary and requiring confirmation in future studies. Future research should be conducted in prospective, multicenter settings with external validation cohorts, incorporate a broader spectrum of cervical cancer histologies, and explore the integration of emerging molecular biomarkers—such as circulating tumor DNA—with existing serum protein markers to construct more precise recurrence prediction models. Furthermore, emerging evidence suggests a potential role for adjuvant HPV vaccination in reducing viral persistence and improving outcomes after treatment for HPV-related disease [39]. While this strategy is primarily studied in the setting of cervical intraepithelial lesions, it represents an intriguing avenue for tertiary prevention in cervical cancer that warrants further investigation in conjunction with refined risk stratification tools like the one proposed in our study.

In conclusion, this retrospective exploratory analysis demonstrates that pretreatment serum levels of SCC, HE4, and TSGF are significantly associated with aggressive tumor characteristics and recurrence risk in cervical cancer patients within the study cohort. Among these, HE4 and the combined index (LogitScore) were identified as potential prognostic predictors independent of clinical stage in this internally validated model. Given that the LogitScore was derived and evaluated within the same cohort without external validation, these findings should be considered exploratory and hypothesis-generating. Further validation in independent external cohorts is necessary before these markers can be recommended for routine clinical practice. This integrated approach could facilitate earlier identification of patients at high risk for recurrence, thereby informing the formulation of individualized adjuvant therapy and intensified surveillance strategies.

## Data Availability

The original contributions presented in the study are included in the article/Supplementary material, further inquiries can be directed to the corresponding author.

## References

[1] Dai D Pei Y Zhu B Wang D Pei S Huang H . Chemoradiotherapy-induced ACKR2+ tumor cells drive CD8+ T cell senescence and cervical cancer recurrence. Cell Rep Med. (2024) 5:101550. doi: 10.1016/j.xcrm.2024.101550, 38723624 PMC11148771

[2] Caruso G Wagar MK Hsu HC Hoegl J Rey Valzacchi GM Fernandes A . Cervical cancer: a new era. Int J Gynecol Cancer. (2024) 34:1946–70. doi: 10.1136/ijgc-2024-005579, 39117381

[3] Cibula D Dostálek L Jarkovsky J Mom CH Lopez A Falconer H . Post-recurrence survival in patients with cervical cancer. Gynecol Oncol. (2022) 164:362–9. doi: 10.1016/j.ygyno.2021.12.018, 34955236 PMC9406127

[4] Hwang WY Suh DH Kim K Kim YB No JH. Serum human epididymis protein 4 as a prognostic marker in cervical cancer. Cancer Control. (2022) 29:10732748221097778. doi: 10.1177/10732748221097778, 35506739 PMC9072869

[5] Hanžek A Siatka C Duc AE. Diagnostic role of urine human epididymis protein 4 in ovarian cancer. Biochem Med. (2024) 34:030502. doi: 10.11613/BM.2024.030502, 39435168 PMC11493460

[6] Xu X Wang W Tian B Zhang X Ji Y Jing J. The predicting role of serum tumor-specific growth factor for prognosis of esophageal squamous cell carcinoma. BMC Cancer. (2023) 23:1067. doi: 10.1186/s12885-023-11602-x, 37932676 PMC10626642

[7] Bønløkke S Stougaard M Blaakær J Bertelsen J Andersen K Fuglsang K . HPV is an essential driver in recurrence of cervical cancer. Pathol Res Pract. (2024) 264:155672. doi: 10.1016/j.prp.2024.155672, 39520972

[8] Tsementzi D Meador R Eng T Shelton J Scott I Konstantinidis KT . Associations among HPV persistence, the vaginal microbiome, and cervical cancer recurrence. J Transl Med. (2025) 23:858. doi: 10.1186/s12967-025-06811-w, 40750891 PMC12317609

[9] Diggs A Huang Y Melamed A Szamreta E Monberg MJ Hershman D . Patterns of use of primary and first-line chemotherapy for recurrence among patients with cervical cancer. Int J Gynecol Cancer. (2024) 34:1001–10. doi: 10.1136/ijgc-2023-004860, 38851239

[10] Mahner S Trillsch F Kwon JS Ferguson SE Bessette P Sebastianelli A . Surgical approach, preoperative LEEP/conization and patterns of recurrence and death in low-risk cervical cancer - exploratory analysis from the CCTG CX.5/SHAPE trial. Int J Surg. (2025) 111:8099–107. doi: 10.1097/JS9.0000000000003027, 40694021 PMC12626566

[11] Lou H Cai H Huang X Li G Wang L Liu F . Cadonilimab combined with chemotherapy with or without bevacizumab as first-line treatment in recurrent or metastatic cervical Cancer (COMPASSION-13): a phase 2 study. Clin Cancer Res. (2024) 30:1501–8. doi: 10.1158/1078-0432.CCR-23-3162, 38372727 PMC11016896

[12] Bruno M Bizzarri N Teodorico E Certelli C Gallotta V Pedone Anchora L . The potential role of systemic inflammatory markers in predicting recurrence in early-stage cervical cancer. Eur J Surg Oncol. (2024) 50:107311. doi: 10.1016/j.ejso.2023.107311, 38056022

[13] Markus M Sartor H Bjurberg M Trägårdh E. Metabolic parameters of [18F]FDG PET-CT before and after radiotherapy may predict survival and recurrence in cervical cancer. Acta Oncol. (2023) 62:180–8. doi: 10.1080/0284186X.2023.2181100, 36815676

[14] Schaafsma M van den Helder R Mom CH Steenbergen RDM Bleeker MCG van Trommel NE. Recurrent cervical cancer detection using DNA methylation markers in self-collected samples from home. Int J Cancer. (2025) 156:659–67. doi: 10.1002/ijc.35143, 39175103 PMC11621989

[15] Ma GF Lin GL Wang ST Huang YY Xiao CL Sun J . Prediction of recurrence-related factors for patients with early-stage cervical cancer following radical hysterectomy and adjuvant radiotherapy. BMC Womens Health. (2024) 24:81. doi: 10.1186/s12905-023-02853-8, 38297248 PMC10829327

[16] Aşıcıoğlu O Ateş S Varol F Yalta TD Sayın C. Oncologic outcome and recurrence patterns of clinical stage IB and IIA cervical cancer: a large retrospective analysis of a tertiary reference center. Int J Gynaecol Obstet. (2026) 172:295–304. doi: 10.1002/ijgo.70366, 40662455 PMC12724023

[17] Di X Gao Z Yu H Liu X Zhao J Wang J . 125I seed brachytherapy for non-central pelvic recurrence of cervical cancer after external beam radiotherapy. Radiat Oncol. (2024) 19:70. doi: 10.1186/s13014-024-02454-1, 38849839 PMC11162001

[18] Shi V Grover S Huang Y Thaker PH Kuroki LM Powell MA . Accuracy of surveillance serum squamous cell carcinoma antigen for cervical cancer recurrence after definitive chemoradiation. Int J Gynecol Cancer. (2024) 34:808–16. doi: 10.1136/ijgc-2024-005303, 38684343 PMC12232631

[19] Wang Y Wang T Yan D Zhao H Wang M Liu T . Vaginal microbial profile of cervical cancer patients receiving chemoradiotherapy: the potential involvement of *Lactobacillus iners* in recurrence. J Transl Med. (2024) 22:575. doi: 10.1186/s12967-024-05332-2, 38886729 PMC11184707

[20] Golia D'Augè T Caruso G Laudani ME Nazzaro L De Vitis LA Rosanu NM . Para-aortic lymph node recurrence in surgically treated early-stage cervical cancer without Para-aortic lymph node surgical staging. Int J Gynecol Cancer. (2024) 34:1867–73. doi: 10.1136/ijgc-2024-005950, 39379329

[21] Pan B Wan T Jiang Y Zheng X Liu P Xiang H . Impact of the initial site of metastases on post-recurrence survival for neuroendocrine cervical cancer. BMC Cancer. (2022) 22:655. doi: 10.1186/s12885-022-09737-4, 35698184 PMC9195210

[22] Hasegawa K Takahashi S Ushijima K Okadome M Yonemori K Yokota H . Cemiplimab monotherapy in Japanese patients with recurrent or metastatic cervical cancer. Cancer Med. (2024) 13:e70236. doi: 10.1002/cam4.70236, 39325020 PMC11426160

