# Supplementary material for: Expression levels of serum SCC, HE4, and TSGF in cervical cancer patients and their correlation with recurrence: a retrospective study
Source: Front Med (Lausanne). 2026 May 11;13:1813161. doi: 10.3389/fmed.2026.1813161 (PMC13199320; doi:10.3389/fmed.2026.1813161)
Supplement: Supplementary file 1 [file Table_1.docx]

Supplementary Table S1. Distribution of FIGO Stage by Initial Treatment Modality

| Initial Treatment Modality | Total  (N=107) | FIGO Stage I-II  (n=69) | FIGO Stage III-IV  (n=38) |
| --- | --- | --- | --- |
| Radical Surgery (± Adjuvant) | 20 (100%) | 18 (90.0%) | 2 (10.0%) |
| Radical RT / CCRT | 72 (100%) | 48 (66.7%) | 24 (33.3%) |
| Systemic Therapy Primarily | 15 (100%) | 0 (0.0%) | 15 (100.0%) |

Note: Data are presented as n (%). Abbreviations: RT, radiotherapy; CCRT, concurrent chemoradiotherapy.
